# Thymic transcriptome analysis after Newcastle disease virus inoculation in chickens and the influence of host small RNAs on NDV replication

**DOI:** 10.1038/s41598-021-89464-1

**Published:** 2021-05-13

**Authors:** Liangxing Guo, Zhaokun Mu, Furong Nie, Xuanniu Chang, Haitao Duan, Haoyan Li, Jingfeng Zhang, Jia Zhou, Yudan Ji, Mengyun Li

**Affiliations:** 1 Henan University of Animal Husbandry and Economy, No. 2 Yingcai Street, Huiji District, Zhengzhou, 450044 China; 2 South China Agricultural University, Guangzhou, 510000 China; 3 Henan Chenxia Biomedical Co., Ltd, Zhengzhou, 450000 China

**Keywords:** Biological techniques, Cell biology, Molecular biology

## Abstract

Newcastle disease (ND), caused by virulent Newcastle disease virus (NDV), is a contagious viral disease affecting various birds and poultry worldwide. In this project, differentially expressed (DE) circRNAs, miRNAs and mRNAs were identified by high-throughput RNA sequencing (RNA-Seq) in chicken thymus at 24, 48, 72 or 96 h post LaSota NDV vaccine injection versus pre-inoculation group. The vital terms or pathways enriched by vaccine-influenced genes were tested through KEGG and GO analysis. DE genes implicated in innate immunity were preliminarily screened out through GO, InnateDB and Reactome Pathway databases. The interaction networks of DE innate immune genes were established by STRING website. Considering the high expression of gga-miR-6631-5p across all the four time points, DE circRNAs or mRNAs with the possibility to bind to gga-miR-6631-5p were screened out. Among DE genes that had the probability to interact with gga-miR-6631-5p, 7 genes were found to be related to innate immunity. Furthermore, gga-miR-6631-5p promoted LaSota NDV replication by targeting insulin induced gene 1 (INSIG1) in DF-1 chicken fibroblast cells. Taken together, our data provided the comprehensive information about molecular responses to NDV LaSota vaccine in Chinese Partridge Shank Chickens and elucidated the vital roles of gga-miR-6631-5p/INSIG1 axis in LaSota NDV replication.

## Introduction

Newcastle disease (ND), caused by virulent Newcastle disease virus (NDV), is a devastating transmissible disease that can affect human and more than 250 avian species (*e.g.* turkeys, chickens, and pigeons) worldwide^1–3^. ND is a major threat to poultry industry and public health throughout the world because of its high incidence and mortality rate^1,2,4^. NDV (a single-stranded RNA virus), also called avian paramyxovirus serotype 1 virus, is a member of the *Avulavirus* genus and *Paramyxoviridae* family^5^. NDV can be divided into 3 major pathotypes (*i.e.* lentogenic, mesogenic and velogenic strains) according to their virulence^2,6^. Lentogenic strain can trigger mild respiratory infection, while velogenic strain can cause hemorrhagic intestinal lesions, respiratory and nervous disorders and high mortality^6,7^. The vaccination of NDV is an effective approach to control ND^8^. Currently, inactivated and live attenuated NDV strains are frequently used as the vaccines to protect poultry and birds against virulent NDV infection^8,9^. Live vaccines are known for their strong protective efficacy by virtue of their capacity to efficiently induce a series of robust immune responses^8,10^. Among these live attenuated NDV vaccine schemes, LaSota strain is the most widely utilized formulation in multiple countries^8,10^. However, “vaccine failure” (*e.g.* improper or incomplete immunization) is a frequently occurred problem that can give rise to the death of poultry post virulent NDV infection^8^.

Recently, non-coding RNAs including circular RNAs (circRNAs) and microRNAs (miRNAs) have attracted much attention from researchers due to their regulatory potential on gene expression in animals including human and poultry^11–14^. CircRNAs are a group of covalently single-stranded closed RNA molecules with little or no protein-coding potential^12,15^. MiRNAs are a class of small non-coding transcripts with the length of about 20 nucleotides (nt)^16^. High-throughput RNA sequencing (RNA-seq), a revolutionized technology with low background signal, can provide the comprehensive transcriptome information of cells, tissues and circulatory system under specific conditions^17,18^. Over the past decades, RNA-seq, function annotation/enrichment and bioinformatics prediction analysis has been used to determine the expression profiles of transcripts (including circRNAs, miRNAs, and mRNAs) and speculate/decipher the functions and regulatory networks of these biomolecules^19–22^.

To better manage the problem of “vaccine failure”, it is imperative to have a deep insight into thymic transcriptome alterations in response to NDV LaSota vaccine treatment and related regulatory networks. In this project, RNA-seq technology was used to identify differentially expressed (DE) circRNAs, miRNAs, and mRNAs at different time intervals (24, 48, 72, 96 h) post NDV LaSota vaccine inoculation in the thymic tissues of Chinese Partridge Shank Chickens. Moreover, Kyoto Encyclopedia of Genes and Genomes (KEGG) and Gene Ontology (GO) enrichment analysis was performed to characterize the functions of DE genes and identify crucial biological processes/pathways/terms in response to vaccine treatment. Additionally, GO, Reactome pathway, and InnateDB databases were utilized to screen out DE genes implicated in innate immunity. Furthermore, bioinformatics analysis was conducted to identify DE circRNAs or mRNAs that had the possibility to interact with a core miRNA. Also, the influence of this central miRNA on NDV replication along with its downstream targets were examined by in vitro cell experiments.

## Results

In this text, differential expression profiles of circRNAs, miRNAs and mRNAs were examined by RNA-seq in chicken thymic tissues at 24 (B), 48 (C), 72 (D) or 96 (E) h after LaSota NDV vaccine inoculation compared to pre-inoculation (A) group, and corresponding results were presented in Supplementary Tables 1, 2 and 3, respectively.

### Identification of DE circRNAs

**Figure 1:**
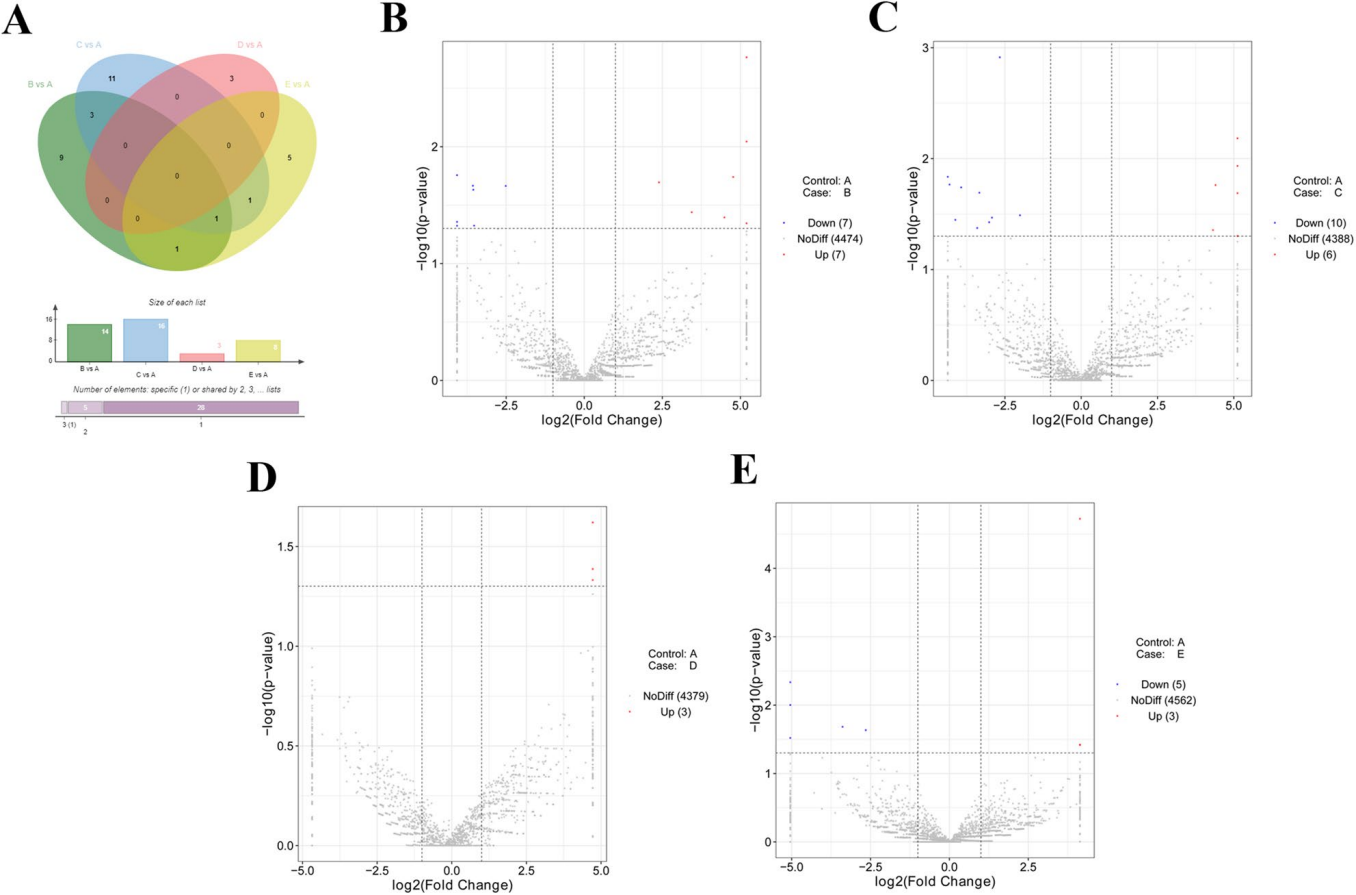
Identification of DE circRNAs. (**A**) Venn diagram of DE circRNAs in B vs A, C vs A, D vs A, and E vs A groups. (**B**–**E**) Volcano plot of circRNA expression profiles in B vs A (**B**), C vs A (**C**), D vs A (**D**), and E vs A (**E**) groups. Venn plot was drawn by jvenn website (http://jvenn.toulouse.inra.fr/app/example.html)^51^.

Among these transcripts, 14, 16, 3 or 8 circRNAs were found to be differentially expressed (|log_2_FoldChange|> 1, *P* value < 0.05) in B versus (vs) A, C vs A, D vs A, or E vs A groups, respectively (Fig. 1A). In detail, 7 circRNAs (ggacirc_033375, ggacirc_030677, ggacirc_028880, ggacirc_022470, ggacirc_015844, ggacirc_010988 and ggacirc_007720) were highly expressed and 7 circRNAs (ggacirc_035788, ggacirc_033324, ggacirc_023318, ggacirc_018072, ggacirc_013281, ggacirc_008182, and ggacirc_007591) were low expressed at 24 h post vaccine treatment relative to pre-treatment group (Supplementary Table 4, Fig. 1B). The expression of 10 circRNAs (ggacirc_005990, ggacirc_007720, ggacirc_010988, ggacirc_015844, ggacirc_016569, ggacirc_022025, ggacirc_028880, ggacirc_030614, ggacirc_033182 and ggacirc_036637) was notably down-regulated and expression of 6 circRNAs (ggacirc_003001, ggacirc_018753, ggacirc_021795, ggacirc_032966, ggacirc_036392 and ggacirc_036448) was markedly up-regulated at 48 h after vaccine injection compared to control group (Supplementary Table 4, Fig. 1C). Moreover, expression of 3 circRNAs (ggacirc_024426, ggacirc_013774 and ggacirc_012028) was strikingly increased at 72 h following vaccine inoculation versus control group (Supplementary Table 4, Fig. 1D). Additionally, 5 circRNAs (ggacirc_022025, ggacirc_034572, ggacirc_010988, ggacirc_001856, and ggacirc_014069) were found to be low expressed and 3 circRNAs (ggacirc_008182, ggacirc_004399 and ggacirc_028358) were demonstrated to be highly expressed at 96 h post vaccine treatment compared with control group (Supplementary Table 4, Fig. 1E). These data suggested that circRNAs were time-dependently expressed after NDV vaccine inoculation in chicken thymic tissues.

### Identification of DE miRNAs

**Figure 2:**
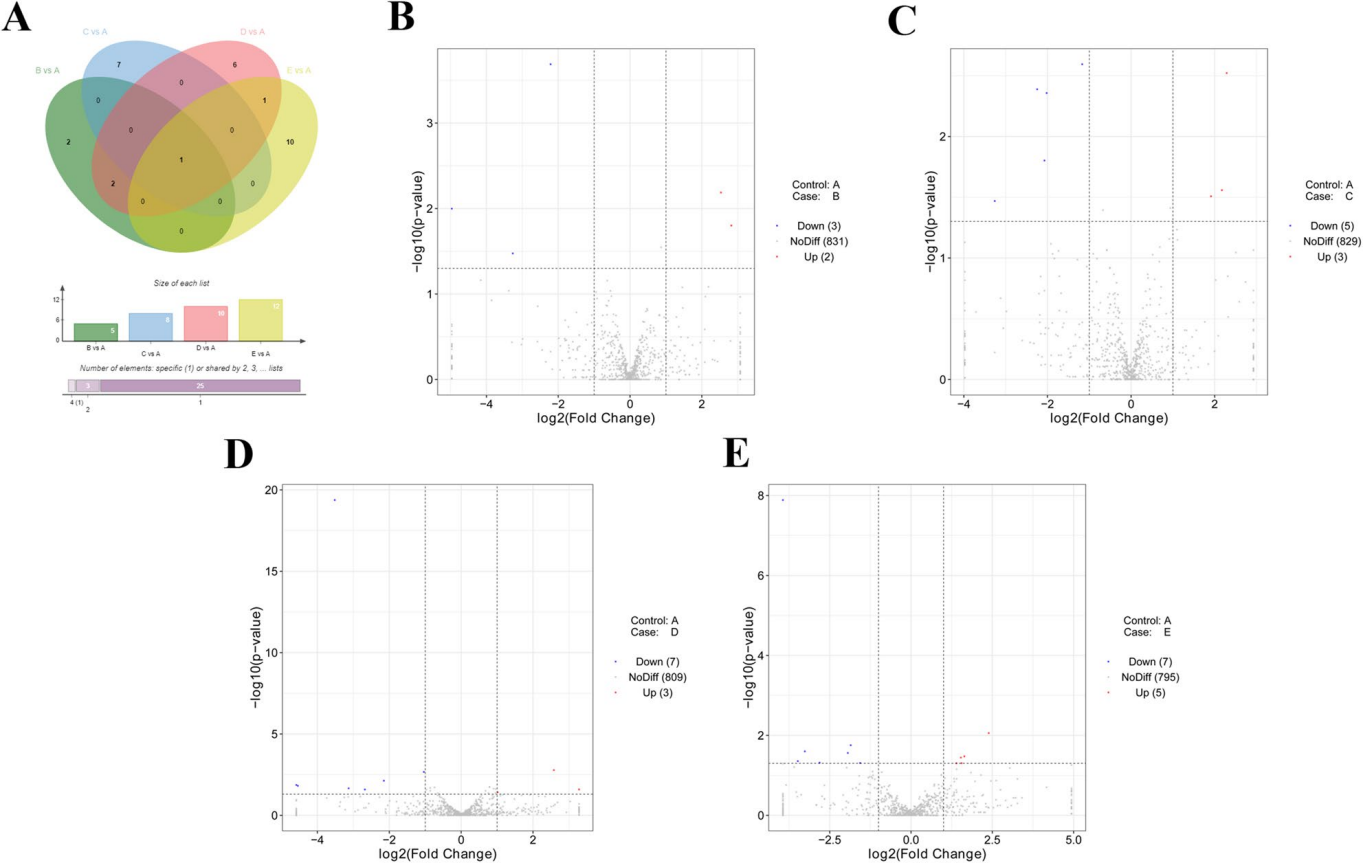
Identification of DE miRNAs. (**A**) Venn diagram of DE miRNAs in B vs A, C vs A, D vs A, and E vs A groups. (**B**–**E**) Volcano plot of miRNA expression patterns in B vs A (**B**), C vs A (**C**), D vs A (**D**), and E vs A (**E**) groups. Venn plot was drawn by jvenn website (http://jvenn.toulouse.inra.fr/app/example.html)^51^.

Moreover, RNA-seq data revealed that 5, 8, 10 or 12 miRNAs were differentially expressed (|log_2_FoldChange|> 1, *P* value < 0.05) in B vs A, C vs A, D vs A, or E vs A group, respectively (Supplementary Table 5, Fig. 2A). In detail, 2 miRNAs (gga-miR-6631-5p and gga-miR-1651-5p) were highly expressed and 3 miRNAs (gga-miR-1551-5p, gga-miR-1798-3p, gga-miR-1682) were low expressed at 24 h post NDV vaccine treatment than that in control group (Supplementary Table 5, Fig. 2B). Moreover, the expression of 3 miRNAs (gga-miR-1727, gga-miR-6575-5p, and gga-miR-6631-5p) was markedly up-regulated and expression of 5 miRNAs (gga-miR-6548-3p, gga-miR-12273-3p, gga-miR-1744-3p, gga-miR-6655-5p and gga-miR-1712-3p) was notably down-regulated at 48 h after vaccine inoculation relative to control group (Supplementary Table 5, Fig. 2C). In addition, 3 up-regulated miRNAs (gga-miR-6631-5p, gga-miR-1784b-5p and gga-miR-196-5p) and 7 down-regulated miRNAs (gga-miR-205b, gga-miR-145-5p, gga-miR-1682, gga-miR-1684a-3p, gga-miR-1551-5p, gga-miR-6670-5p and gga-miR-1453) were found in D vs A group (Supplementary Table 5, Fig. 2D). Furthermore, 5 miRNAs (gga-miR-99a-5p, gga-miR-1434, gga-miR-148a-3p, gga-miR-2131-3p and gga-miR-6631-5p) were highly expressed and 7 miRNAs (gga-miR-205b, gga-miR-1329-3p, gga-miR-6586-5p, gga-miR-449c-3p, gga-miR-34c-3p, gga-miR-1677-5p and gga-miR-129–1-3p) were low expressed in thymic tissues of chickens at 96 h post-inoculation in comparison with control group (Supplementary Table 5, Fig. 2E). Additionally, we noticed that the expression of gga-miR-6631-5p was significantly up-regulated across all the time points (24, 48, 72, or 96 h) post vaccine treatment relative to control group (Fig. 2A), suggesting that gga-miR-6631-5p might play vital roles in the responses to vaccine.

### Identification of DE mRNAs

**Figure 3:**
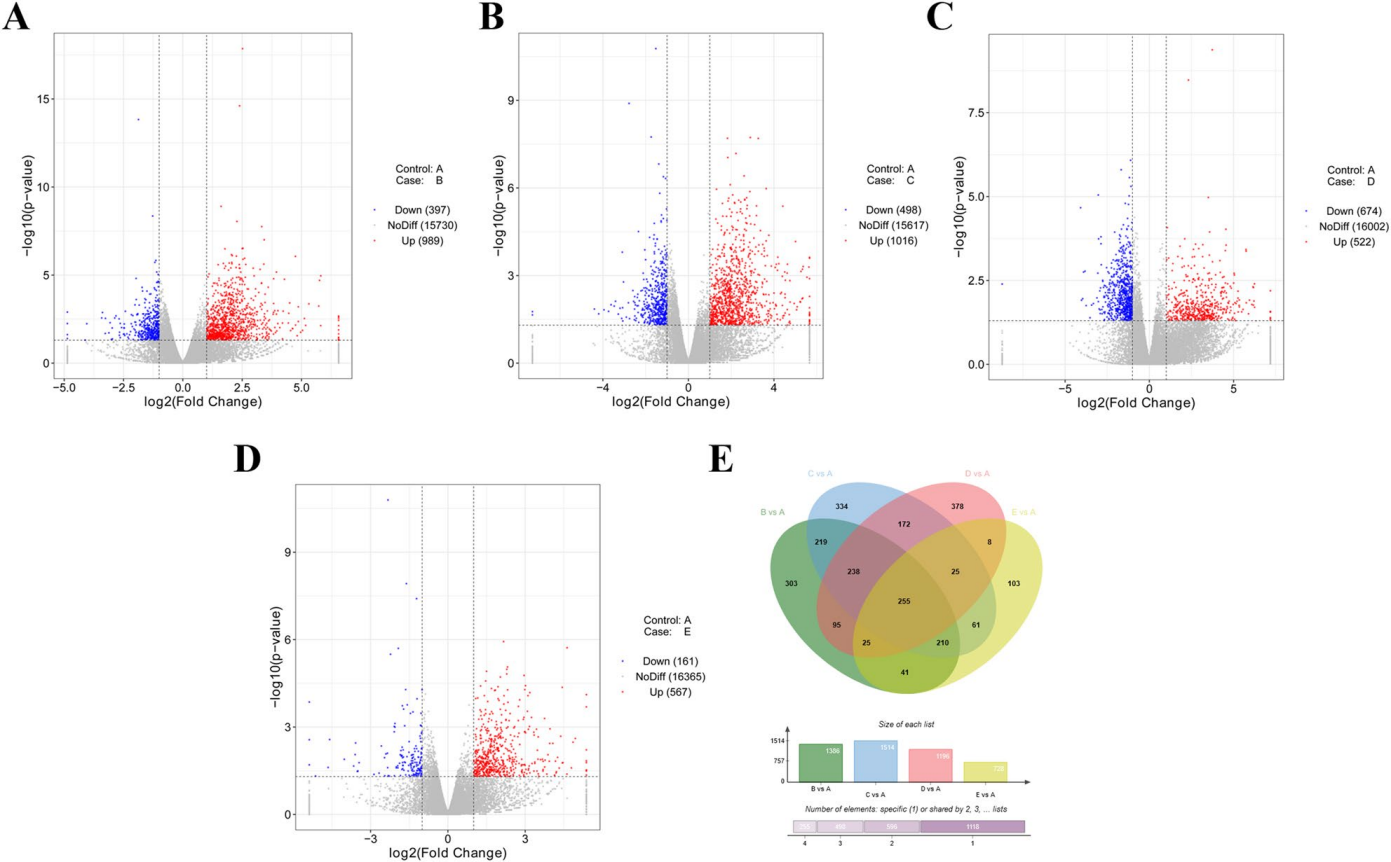
Identification of DE mRNAs. (**A**–**D**) Volcano plot of miRNA expression in B vs A (**A**), C vs A (**B**), D vs A (**C**), and E vs A (**D**) groups. (**E**) Venn diagram of DE mRNAs in B vs A, C vs A, D vs A, and E vs A groups. Venn plot was drawn by jvenn website (http://jvenn.toulouse.inra.fr/app/example.html)^51^.

In addition, 989 up-regulated mRNAs and 397 down-regulated mRNAs were found in chicken thymus tissue samples at 24 h post vaccine injection relative to control group (Supplementary Table 6, Fig. 3A). The expression levels of 1016 mRNAs were notably up-regulated and expression levels of 498 mRNAs were markedly down-regulated in C vs A group (Supplementary Table 7, Fig. 3B). Also, 522 mRNAs were found to be highly expressed and 674 mRNAs were demonstrated to be low expressed in D vs A group (Supplementary Table 8, Fig. 3C). Moreover, 728 mRNAs (567 up-regulated and 161 down-regulated) were dysregulated at 72 h post vaccine treatment compared to control group (Supplementary Table 9, Fig. 3D). Furthermore, 255 differentially expressed mRNAs were identified across all the time points (24, 48, 72, and 96 h) after vaccine inoculation versus control group (Supplementary Table 10, Fig. 3E).

### Enrichment analysis for DE mRNAs

**Figure 4:**
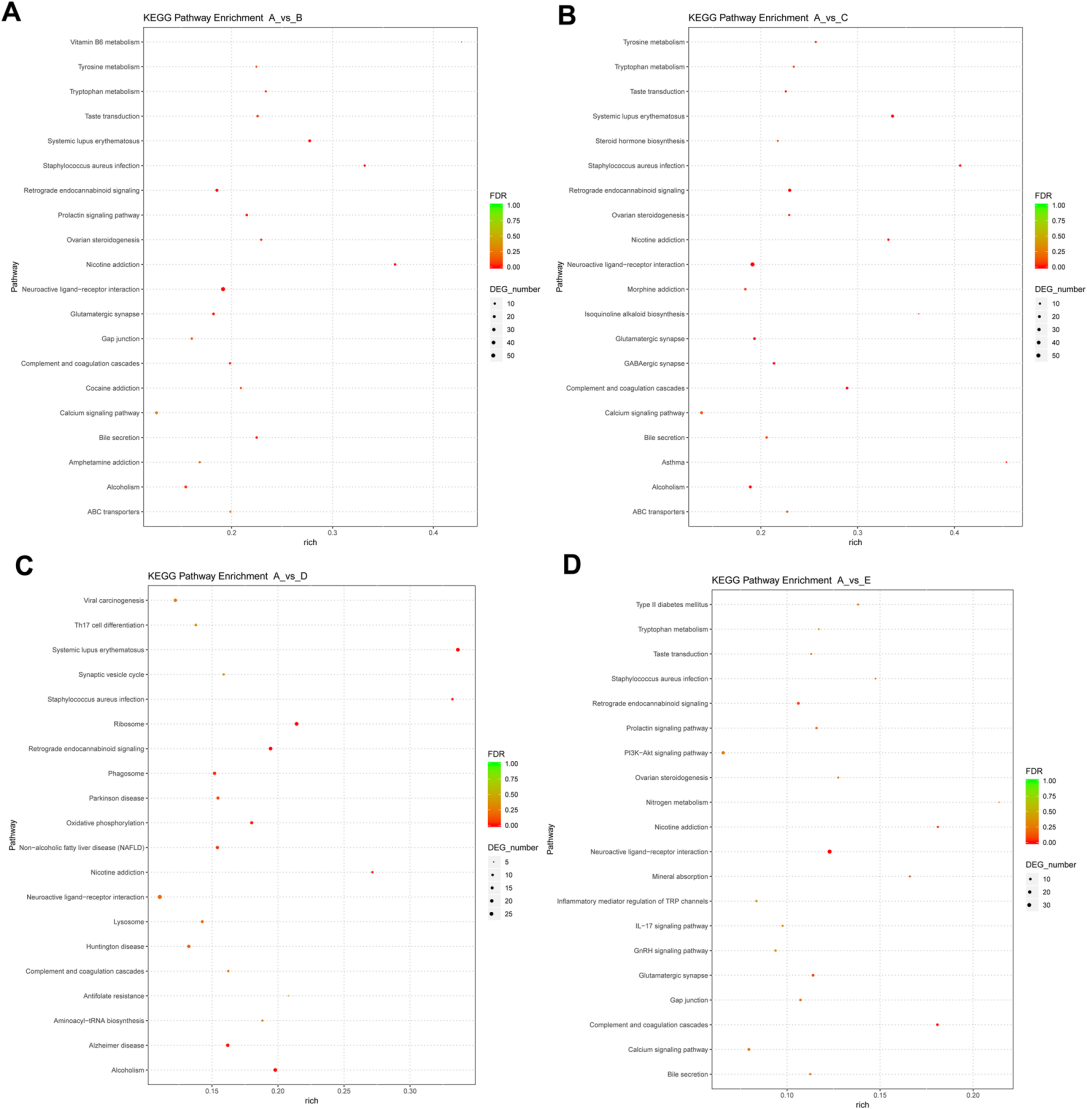
KEGG enrichment analysis for DE genes. (**A**–**D**) Top 20 KEGG pathways enriched by DE genes in B vs A, C vs A, D vs A, and E vs A groups, respectively. Pathway in the Y-axis represents the KEGG pathway term enriched by differentially expressed genes. The “rich” in the X-axis represents the rich factor. Rich factor: the number of differentially expressed genes in the pathway term/the number of all annotated genes in the pathway term. FDR: P value after the correction of False Discovery Rate (FDR). DEG_number: number of differentially expressed genes in the KEGG pathway term. KEGG enrichment analysis was performed as previously described^48–50^.

Next, KEGG and GO enrichment analysis for DE mRNAs was conducted to examine the crucial terms/pathways in the responses to NDV vaccine. The top 20 KEGG pathways enriched by DE genes in B vs A, C vs A, D vs A, or E vs A group were presented in Fig. 4A–D, respectively (Supplementary Table 11). GO annotation analyses showed that 19 (Supplementary Table 12 sheet 2), 18 (Supplementary Table 13 sheet 2), 21 (Supplementary Table 14 sheet 2) or 7 (Supplementary Table 15 sheet 2) biological process terms were significantly enriched by the DE genes in B vs A, C vs A, D vs A, or E vs A group, respectively. These biological processes or pathways might function as crucial players in the responses to NDV vaccine.

### Screening of vaccine-influenced innate immune genes

Additionally, 32 genes were found to be implicated in innate immune responses by seeking for the terms containing the key word of innate immune in GO annotation data (Supplementary Table 12–15, sheet 1) for DE genes in B vs A, C vs A, D vs A and E vs A groups. The information of these genes was integrated in Supplementary Table 16. Genes with known names were nuclear receptor subfamily 1 group H member 4 (NR1H4), RNA binding motif protein 14 (RBM14), solute carrier family 26 member 6 (SLC26A6), solute carrier family 11 member 1 (SLC11A1), mannan binding lectin serine peptidase 2 (MASP2), cytochrome b-245 alpha chain (CYBA), cathelicidin-B1-like (CATHB1), pentraxin 3 (PTX3), mannan binding lectin serine peptidase 1 (MASP1), collectin subfamily member 11 (COLEC11), suppressor of cytokine signaling 1-like protein (SOCS1L), otopetrin 1 (OTOP1), cochlin (COCH), transmembrane protein 173 (TMEM173), complement factor D (CFD), apolipoprotein A4 (APOA4), macrophage migration inhibitory factor (glycosylation-inhibiting factor) (MIF), retinoic acid receptor responder (tazarotene induced) 2 (RARRES2), tripartite motif containing 62 (TRIM62), triokinase and FMN cyclase (TKFC), serpin family G member 1 (SERPING1), hexamethylene bisacetamide inducible 1 (HEXIM1), glyceraldehyde-3-phosphate dehydrogenase (GAPDH), signal transducer and activator of transcription 2 (STAT2), FAU, ubiquitin like and ribosomal protein S30fusion (FAU), NOP53 ribosome biogenesis factor (NOP53), growth factor independent 1 transcriptional repressor (GFI1) and NLR family member X1 (NLRX1) (Supplementary Table 16 sheet 2). Considering the conservation of innate immune responses among different organisms, more innate immunity-related genes were screened out from InnateDB and Reactome Pathway databases.

Expression patterns of 712 innate immune genes in the InnateDB database (http://innatedb.sahmri.com/annotatedGenes.do?type=innatedb, Supplementary Table 17) were analyzed in our experiments (Supplementary Table 18, sheet 1). Among these innate immune genes annotated by InnateDB database, 48 (24 up-regulated and 24 down-regulated) (Supplementary Table 18, sheet 2), 50 (19 up-regulated and 31 down-regulated) (Supplementary Table 18, sheet 3), 60 (14 up-regulated and 46 down-regulated) (Supplementary Table 18, sheet 4) or 21 (13 up-regulated and 8 down-regulated) (Supplementary Table 18, sheet 5) genes were found to be differentially expressed in B vs A, C vs A, D vs A or E vs A group, respectively. Moreover, 4 innate immune genes (CCAAT enhancer binding protein beta (CEBPB), NFKB inhibitor beta (NFKBIB), granulin precursor (GRN) and frizzled class receptor 1 (FZD1)) were markedly down-regulated and 6 innate immune genes (amphiregulin (AREG), complement C8 alpha chain (C8A), interleukin 22 (IL22), GATA binding protein 4 (GATA4), interleukin 1 receptor accessory protein like 1 (IL1RAPL1), and coagulation factor XI (F11)) were notably up-regulated in all the groups of B vs A, C vs A, D vs A, and E vs A groups (Supplementary Table 18, sheet 6).

Additionally, expression patterns of 319 innate immune genes in the Reactome Pathway database (http://reactome.ncpsb.org/PathwayBrowser/#/R-GGA-168249&PATH=R-GGA-168256&DTAB=MT) (Supplementary Table 19) were examined in our experiments (Supplementary Table 20, sheet 1). Among these genes, 15 (11 up-regulated and 4 down-regulated) (Supplementary Table 20, sheet 2), 15 (11 up-regulated and 4 down-regulated) (Supplementary Table 20, sheet 3), 20 (7 up-regulated and 13 down-regulated) (Supplementary Table 20, sheet 4) or 7 (7 up-regulated) (Supplementary Table 20, sheet 5) genes were found to be differentially expressed in B vs A, C vs A, D vs A, or E vs A group, respectively. Furthermore, 3 common innate immunity-related genes (complement factor I (CFI), complement C8 alpha chain (C8A), and fyn related Src family tyrosine kinase (FRK)) were found to be strikingly up-regulated in all the groups of B vs A, C vs A, D vs A and E vs A (Supplementary Table 20 sheet 6). The information of these innate immunity-associated DE genes annotated by GO, Reactome pathway, and InnateDB databases was integrated into Supplementary Table 21.

### Interaction networks of screened innate immune genes

**Figure 5:**
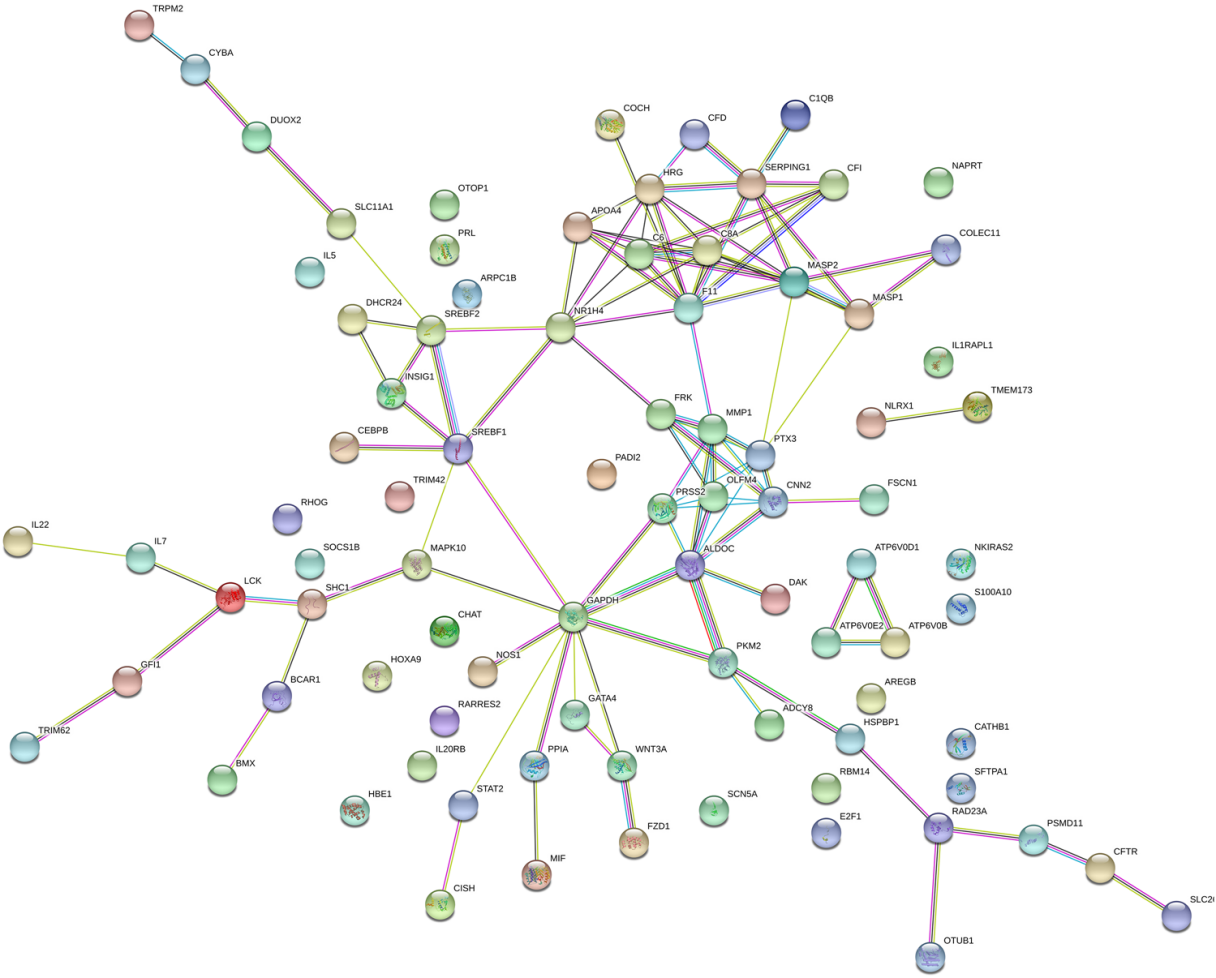
Potential interaction networks of proteins encoded by innate immune genes. The interaction networks were drawn using the STRING: functional protein association networks (https://string-db.org/).

Next, the interactions among proteins encoded by DE innate immune genes were explored through STRING: functional protein association networks (https://string-db.org/). We noticed that 86 items could be found among 96 screened innate immune genes with known names (7 IDs without known names) in STRING website (https://string-db.org/cgi/network.pl?taskId=da9LIhlXqdF5). The image of interaction network was presented in Fig. 5. The interaction edges with the combined score ≥ 0.4 were shown in sheet 1 of Supplementary Table 22. The annotation information of these proteins was provided in sheet 2 of Supplementary Table 22. KEGG analysis showed that 9 pathways, including Phagosome pathway, NOD-like receptor signaling pathway, RIG-I-like receptor signaling pathway, Glycolysis/Gluconeogenesis and Cytokine-cytokine receptor interaction pathways, were significantly enriched by these innate immune genes (Supplementary Table 23). In detail, 6 Phagosome pathway related proteins (ATPaseV0 subunit b (ATP6V0B), V-type proton ATPase subunit (ATP6V0E2), COLEC11, cytochrome b-245 light chain (CYBA), Nitric oxide synthase 1 (NOS1), surfactant, pulmonary-associated protein A1 (SFTPA1)), 6 NOD-like receptor signaling pathway related proteins (CYBA, mitogen-activated protein kinase 10 (MAPK10), NLR family member X1 (NLRX1), signal transducer and activator of transcription 2 (STAT2), TMEM173, transient receptor potential cation channel, subfamily M, member 2 (TRPM2)), 4 RIG-I-like receptor signaling pathway related proteins (Triokinase and FMN cyclase (TKFC/DAK),MAPK10,NLRX1,TMEM173), 3 Glycolysis/Gluconeogenesis-related proteins (aldolase C fructose-bisphosphate (ALDOC), Glyceraldehyde-3-phosphate dehydrogenase (GAPDH), pyruvate kinase 2 (PKM2)), and 5 Cytokine-cytokine receptor interaction related proteins (interleukin 20 receptor beta (IL20RB), interleukin 22 (IL22), interleukin 5 (IL5),interleukin 7 (IL7), prolactin (PRL)) were found in our network (Supplementary Table 23).

### Bioinformatics analysis of circRNAs and gga-miR-6631-5p

Recent studies showed that some circRNAs could exert their regulatory roles through serving as miRNA sponges^23,24^. CircRNA-miRNA pairs were predicted by miRanda software. Considering the abnormal expression of gga-miR-6631-5p across all the time points (24, 48, 72, or 96 h) post NDV vaccine inoculation, we further investigated potential interaction pairs of circRNAs and gga-miR-6631-5p. As presented in sheet 1 of Supplementary Table 24, 1057 possible circRNA/gga-miR-6631-5p pairs were forecasted by miRanda software. Also, expression patterns of these circRNAs in B vs A, C vs A, D vs A and E vs A groups were displayed in sheet 2 of Supplementary Table 24. Combined with circRNA expression data and prediction data, 6 circRNAs (ggacirc_023318, ggacirc_013281, ggacirc_033324, ggacirc_007720, ggacirc_028880 and ggacirc_033375) in B vs A group (Supplementary Table 24 sheet 3), 8 circRNAs (ggacirc_003001, ggacirc_036392, ggacirc_005990, ggacirc_022025, ggacirc_018753, ggacirc_033182, ggacirc_007720 and ggacirc_028880) in C vs A group (Supplementary Table 24 sheet 4), 1 circRNA (ggacirc_013774) in D vs A group (Supplementary Table 24 sheet 5) and 2 circRNAs (ggacirc_022025 and ggacirc_034572) in E vs A group (Supplementary Table 24 sheet 6) were found to be differentially expressed after NDV vaccine injection.

### Prediction of gga-miR-6631-5p potential targets

It is well known that miRNAs can regulate the expression of protein-coding genes at post-transcriptional levels^16^. Prediction analysis showed that 1431 genes had the potential gga-miR-6631-5p binding sites (Supplementary Table 25 sheet 1). Among these possible targets, expression profiles of 1426 items were analyzed in our experiments (Supplementary Table 25 sheet 2). Our outcomes presented that mRNA levels of 14 potential targets were notably down-regulated and mRNA levels of 50 potential targets were markedly up-regulated in B vs A group (Supplementary Table 25 sheet 3). And, 13 down-regulated targets and 60 up-regulated targets were found in C vs A group (Supplementary Table 25 sheet 4). Moreover, 64 targets (30 down-regulated and 34 up-regulated) or 33 targets (5 down-regulated and 28 up-regulated) were demonstrated to be dysregulated in D vs A or E vs A groups, respectively (Supplementary Table 2525 sheet 5 and sheet 6). In addition, 1 target was low expressed and 8 targets were highly expressed in all the groups (Supplementary Table 25 sheet 7). Among these DE targets, 5 genes (E2F1, INSIG1, HSPBP1, MAPK10, MASP2) in B vs A group, 5 genes (SERPING1, E2F1, HSPBP1, MAPK10, MASP2) in C vs A group, 4 genes (E2F1, HSPBP1, PSMD11, INSIG1) in D vs A group and 3 genes (SERPING1, MAPK10, MASP2) in E vs A group were found to be implicated in innate immune responses by scanning the innate immune genes in Supplementary Table 21. Expression data of E2F1, INSIG1, HSPBP1, MAPK10, MASP2, SERPING1 and PSMD11 were presented in Supplementary Table 26.

### MiR-6631-5p overexpression promoted LaSota NDV replication in DF-1 cells

**Figure 6:**
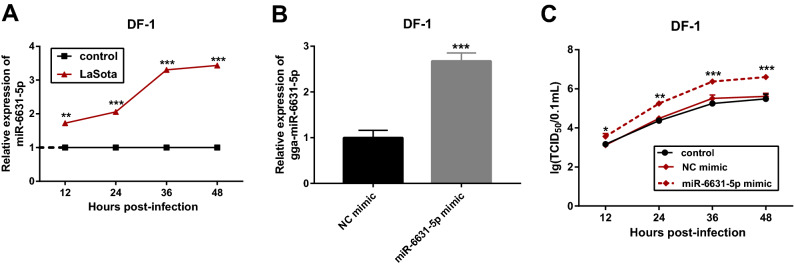
MiR-6631-5p overexpression promoted LaSota NDV replication in DF-1 cells. (A) The level of gga-miR-6631-5p was measured by RT-qPCR assay in DF-1 cells infected with or without LaSota NDV at the indicated time points after infection. (B) DF-1 cells were transfected with gga-miR-6631-5p mimic (100 nM) or NC mimic (100 nM). At 48 h after transfection, gga-miR-6631-5p level was determined by RT-qPCR assay. (C) DF-1 cells were transfected with gga-miR-6631-5p mimic (100 nM) or NC mimic (100 nM). Non-transfected cells acted as the control group. At 24 h after transfection, cells were infected with LaSota NDV at the MOI of 1. At the indicated time points (12, 24, 36, 48 h) after LaSota infection, TCID_50_ assay was performed to examine the effect of gga-miR-6631-5p overexpression on LaSota NDV replication.

Considering the high expression of gga-miR-6631-5p in thymic tissues of Chinese Partridge Shank Chickens across all four time points (24, 48, 72, and 96 h) post NDV vaccine treatment, we supposed that gga-miR-6631-5p might play vital roles in the responses to LaSota NDV. RT-qPCR assay also validated that gga-miR-6631-5p was highly expressed in LoSota-infected DF-1 cells compared to uninfected cells (Fig. 6A), which was in line with the RNA-seq data. To further explore the effect of gga-miR-6631-5p on LaSota NDV replication and related molecular basis, gga-miR-6631-5p mimic and its control NC mimic were synthesized. Transfection efficiency analysis presented that the transfection of gga-miR-6631-5p mimic led to the notable up-regulation of gga-miR-6631-5p level in DF-1 cells than that in cells transfected with NC mimic (Fig. 6B), suggesting that gga-miR-6631-5p mimic could be used for subsequent gain-of-function experiments. Next, the effect of gga-miR-6631-5p overexpression on LaSota NDV replication was further examined by TCID_50_ assay. Results showed that the enforced expression of gga-miR-6631-5p facilitated LaSota NDV replication in DF-1 cells (Fig. 6C).

### The introduction of gga-miR-6631-5p inhibitor hindered LaSota NDV replication by up-regulating INSIG1 in DF-1 cells

**Figure 7:**
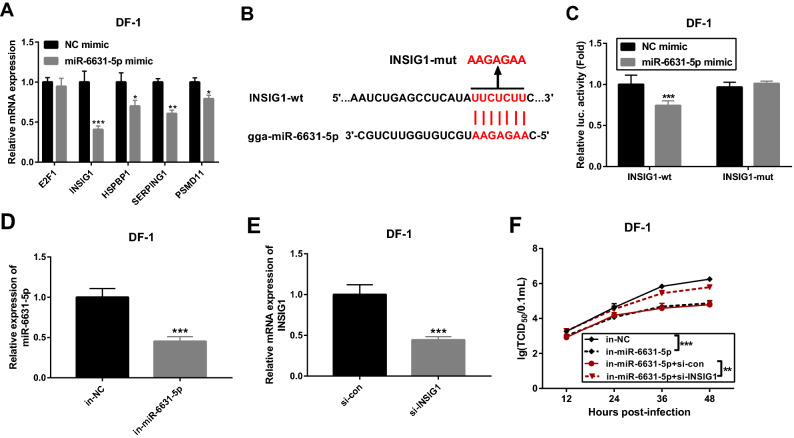
The introduction of gga-miR-6631-5p inhibitor hindered LaSota NDV replication by up-regulating INSIG1 in DF-1 cells. (**A**) The effect of gga-miR-6631-5p overexpression on E2F1, INSIG1, HSPBP1, SERPING1 and PSMD11 mRNA expression was tested by RT-qPCR assay in DF-1 cells at 48 h after transfection. (**B**) Putative complementary sites between gga-miR-6631-5p and INSIG1 3’UTR, and mutant sites in INSIG1-mut reporter. (**C**) DF-1 cells were co-transfected with NC mimic or gga-miR-6631-5p mimic and INSIG1-wt reporter or INSIG1-mut reporter, followed by the detection of luciferase activities at 48 h after transfection. (**D**) The level of gga-miR-6631-5p was measured by RT-qPCR assay at 48 h post transfection in DF-1 cells transfected with in-miR-6631-5p or in-NC. (**E**) DF-1 cells were transfected with si-con or si-INSIG1. Forty-eight hours later, INSIG1 mRNA level was measured through RT-qPCR assay. (**F**) DF-1 cells were transfected with in-NC (100 nM), in-miR-6631-5p (100 nM), in-miR-6631-5p (100 nM) + si-con (100 nM), or in-miR-6631-5p (100 nM) + si-INSIG1 (100 nM). Non-transfected cells acted as the control group. At 24 h after transfection, cells were infected with LaSota NDV at the MOI of 1. Next, TCID_50_ assay was performed at the indicated time points (12, 24, 36, 48 h) after LaSota infection.

As mentioned above, E2F1, INSIG1, HSPBP1, MAPK10, MASP2, SERPING1 and PSMD11 have been found to be related with innate immune responses and to be potential targets of gga-miR-6631-5p. Considering the negative regulatory effects of miRNAs on their targets and gga-miR-6631-5p up-regulation in response to LaSota NDV infection, 5 down-regulated RNAs (E2F1, INSIG1, HSPBP1, SERPING1 and PSMD11) were selected for further investigations. RT-qPCR assay revealed that gga-miR-6631-5p overexpression led to the remarkable down-regulation of INSIG1, HSPBP1, SERPING1 and PSMD11 mRNA levels, but had no much effect on E2F1 mRNA expression (Fig. 7A). In view of the strongest inhibitory effect of gga-miR-6631-5p on INSIG1 mRNA expression, luciferase reporter assay was performed to further explore whether INSIG1 was a target of gga-miR-6631-5p in DF-1 cells. Results showed that gga-miR-6631-5p overexpression led to the notable down-regulation of luciferase activity of INSIG1-wt reporter, but had no much influence on luciferase activity of INSIG1-mut reporter with mutant gga-miR-6631-5p binding site (Fig. 7B,C). These data revealed that gga-miR-6631-5p could bind with INSIG1 3’UTR by predicted sites. Transfection efficiency analysis revealed that the transfection of in-miR-6631-5p could notably reduce gga-miR-6631-5p level in DF-1 cells compared with in-NC control group (Fig. 7D). The transfection of si-INSIG1 led to a dramatic decrease in INSIG1 mRNA expression in DF-1 cells relative to si-con control group (Fig. 7E). TCID_50_ assay revealed that the depletion of gga-miR-6631-5p led to a noticeable reduction in LaSota NDV replicative potential in DF-1 cells (Fig. 7F). In addition, INSIG1 knockdown notably abrogated the detrimental effect of gga-miR-6631-5p loss on LaSota NDV replication in DF-1 cells (Fig. 7F). In a word, these outcomes suggested that INSIG1 was a target of gga-miR-6631-5p and gga-miR-6631-5p promoted LaSota NDV replication through down-regulating INSIG1 in DF-1 cells.

## Discussion

In the present study, we demonstrated that 14, 16, 3 or 8 circRNAs were differentially expressed in thymic tissues of chickens at 24 h, 48 h, 72 h or 96 h after NDV LaSota vaccine inoculation relative to pre-inoculation group. Moreover, 5, 8, 10 or 12 DE miRNAs were identified in B vs A, C vs A, D vs A, or E vs A group, respectively. Furthermore, 1386, 1514, 1196, or 728 mRNAs were found to be differentially expressed in B vs A, C vs A, D vs A, or E vs A group, respectively.

Additionally, 32 DE genes were found to be implicated in innate immune responses based on the GO annotation data. Considering the conservation of innate immune responses among different organisms^25^, more innate immunity-related genes were screened out through InnateDB and Reactome Pathway databases. Integration analysis showed that a total of 103 innate immune genes annotated by GO, Reactome pathway, and InnateDB databases were differentially expressed in response to NDV infection. Among these innate immunity genes, 4 genes (CEBPB, NFKBIB, GRN and FZD1) were low expressed and 8 genes (CFI, C8A, FRK, AREG, IL22, GATA4, IL1RAPL1 and F11) were highly expressed in all the groups of B vs A, C vs A, D vs A, and E vs A. AREG, a member of the epidermal growth factor (EGF) family, has been found to be expressed by multiple innate immune cell populations (*e.g.* basophils, mast cells, neutrophils) and adaptive immune cell populations (CD4^+^ T cells, CD8^+^ T cells) besides epithelial and mesenchymal cells^26^. Moreover, AREG was reported to be involved in mediating pathogen resistance, orchestrating tissue repair and homeostasis, inhibiting local inflammation and regulating immune responses^26^. Qiao et al*.* demonstrated that GATA4 loss inhibited inflammatory responses and NF-κB activation induced by lipopolysaccharide in human dental pulp cells^27^. Also, Kang et al*.* presented that GATA4 was involved in the regulation of inflammatory responses^28^.

Furthermore, the interaction network of proteins encoded by DE innate immune genes was established through STRING website. KEGG enrichment analyses showed that these DE innate immune genes mainly participated in the regulation of 9 KEGG pathways including nucleotide-binding oligomerization domain-containing (NOD)-like receptor signaling pathway, retinoic acid-inducible gene I (RIG-I)-like receptor signaling pathway, cytokine-cytokine receptor interaction and phagosome pathway. Some researchers proposed that phagosome might function as the link organelle between innate and adaptive immunity^29,30^, suggesting the vital roles of phagosome pathway in immune responses. It has been well documented that RNA virus can be recognized by Toll-like receptors, RIG-I-like receptors and NOD-like receptors, whose activation in response to viral RNAs can initiate innate immune responses such as pro-inflammatory cytokine secretion and type I interferon (IFN) production^31–33^. Moreover, Fournier et al*.* pointed out that the intranasal application of NDV MTH68 strain could induce pro-inflammatory cytokine secretion in mouse bronchial lavage fluid and activate RIG-I and IFN pathways in mouse dendritic cells^34^. In addition, previous studies showed that RIG-I and IFN pathways played crucial roles in protecting host cells from NDV infection^35,36^.

Despite the discovery of multitudinous circRNAs over the past decades, the functions and regulatory mechanisms of most circRNAs remain unknown^37^. Recently, accumulating evidence shows that circRNAs can function as miRNA sponges (ceRNAs) to relieve the inhibitory activity of miRNAs on target mRNAs by sequestering miRNAs^38,39^. Considering the abnormal expression of gga-miR-6631-5p across all the time points post NDV vaccine infection, circRNAs or mRNA targets that had a possibility to interact with gga-miR-6631-5p were predicted using the miRanda software. Combined with expression data of circRNAs/mRNAs and prediction data of circRNAs/mRNAs and gga-miR-6631-5p, 6 DE circRNAs and 64 DE genes in B vs A group, 8 DE circRNAs and 73 DE genes in C vs A group, 1 DE circRNA and 64 DE genes in D vs A group, and 2 DE circRNAs and 33 DE genes in E vs A group were found to have the possibility to bind to gga-miR-6631-5p. Among these DE gga-miR-6631-5p targets, 5 genes (E2F1, INSIG1, HSPBP1, MAPK10, MASP2) in B vs A group, 5 genes (SERPING1, E2F1, HSPBP1, MAPK10, MASP2) in C vs A group, 4 genes (E2F1, HSPBP1, PSMD11, INSIG1) in D vs A group and 3 genes (SERPING1, MAPK10, MASP2) in E vs A group were found to be implicated in innate immune responses.

Moreover, our data revealed that gga-miR-6631-5p expression was markedly up-regulated in DF-1 cells infected with LaSota NDV virus compared with uninfected cells. Enforced expression of gga-miR-6631-5p facilitated LaSota NDV replication through targeting INSIG1 in DF-1 cells. INSIG1 has been found to be a bridge in the activation of TANK-binding kinase 1 (TBK1) by promoting K27-linked polyubiquitination of stimulator of interferon genes (STING) and INSIG1 loss curbed STING-mediated antiviral gene induction^40^. TBK1 was a vital kinase in the induction of antiviral innate immune responses^41^ and STING was a negative regulator in the replication of multiple RNA viruses such as Vesicular Stomatitis virus and Sindbis virus^42^. In addition, Ran et al*.* pointed out that STING knockdown suppressed the expression of interferon regulatory factor 7 (IRF7), type I interferon (IFN)-α, and IFN-β in chicken embryo fibroblasts^43^.

The previous transcriptome analyses in embryos^44^, spleen^45^ or trachea^46^ from Leghorn and Fayoumi chickens and chicken embryo fibroblasts^47^ mainly explored the influence of NDV vaccine treatment on immune cells, molecular pathways, and gene expression. In our project, we investigated the expression profile alterations of circRNAs, miRNAs and mRNAs in thymic tissues of Chinese Partridge Shank Chickens at 24, 48, 72, 96 h post NDV infection versus pre-inoculation group. In addition, we further analyzed the expression patterns of 712 innate immune genes annotated by InnateDB database and 319 innate immune genes annotated by Reactome Pathway database in thymic tissues of Chinese Partridge Shank Chickens. A total of 103 innate immune genes annotated by GO, Reactome pathway, and InnateDB databases were identified to be differentially expressed in response to NDV infection in our study. Moreover, we established the potential interaction network of proteins encoded by these dysregulated innate immune genes through String website. Additionally, some possible circRNAs/gga-miR-6631-5p and gga-miR-6631-5p/genes pairs were constructed based on bioinformatics prediction analysis. Furthermore, our data revealed that gga-miR-6631-5p promoted LaSota NDV replication by targeting INSIG1 in DF-1 cells. These data might contribute to the investigation on interactions of NDV LaSota vaccine and Chinese Partridge Shank chickens and the development of new strategies for improving LaSota vaccine efficacy, which has vital values in NDV vaccine immunization work for Chinese native chickens.

## Conclusion

In summary, our data provided the time-specific comprehensive information of circRNAs, miRNAs and mRNAs in response to NDV LaSota vaccine infection in thymic tissues of Chinese Partridge Shank chickens and elucidated the vital roles of gga-miR-6631-5p/INSIG1 axis in LaSota NDV replication. Also, multiple innate immune genes implicated in NDV LaSota vaccine infection were screened out.

## Materials and methods

### Animal experiment

All experimental procedures were approved by Institutional Animal Care and Use Committee of Henan University of Animal Husbandry and Economy. Specific pathogen-free (SPF) Chinese Partridge Shank Chickens (30-days-old) free of NDV antibody were reared in the rooms at the biosafety level II facility. These chickens were inoculated with or without 10^5^ 50% egg-infectious dose (EID_50_) of LaSota NDV vaccine (200 μL/chicken, China Institute of Veterinary Drug Control, Beijing, China) through an ocular route. Chickens were anesthetized and euthanized by the intravenous injection of pentobarbital sodium solution (30 mg/kg body weight), followed by the collection of thymic tissue samples from three chickens at the indicated time point (0, 24, 48, 72 or 96 h) after NDV vaccine injection. Animal experiments were conducted at Animal Laboratory Center of Henan University of Animal Husbandry and Economy. All in vivo experiments were carried out in compliance with the ARRIVE guidelines (http://www.nc3rs.org.uk/page.asp?id=1357).

### Sample preparation and RNA-seq experiment procedures

Total RNA was extracted from thymic tissues using Trizol Reagent (Thermo Scientific, Rockford, IL, USA) and quantified using NanoDrop 2000 spectrophotometer (Thermo Scientific). Moreover, the quality of isolated RNA was analyzed on Agilent Bioanalyzer 2100 (Agilent Technologies, Santa Clara, CA, USA). High-quality RNA samples (OD260/280: 1.8–2.2, RNA concentration ≥ 400 ng/μl, RNA Integrity number ≥ 8) were selected for subsequent experiments. cDNA libraries for mRNAs and circRNAs were constructed using the TruSeq Stranded Total RNA Library Prep Kit (Illumina, San Diego, CA, USA). Small RNA libraries were conducted using the NEBNext Multiplex Small RNA Library Prep Set for Illumina (New England Biolabs, Ipswich, MA, USA) based on the manufacturer’s protocols. After quantified and qualified by Agilent Bioanalyzer 2100 (Agilent Technologies), signal strand cDNA libraries were sequenced on IlluminaHiSeq 2500 instrument (Illumina) through Shanghai Personal Biotechnology Co. Ltd. (Shanghai, China).

### Data processing

Raw sequencing data were analyzed and filtered to obtain high-quality clean data, which were then aligned to the reference genome of Gallus_gallus.Gallus_gallus-5.0 (assembly GCA_000002315.3) using Tophat2.

### Differential expression analysis

Expression analysis was conducted using the method of fragments per kilobase of transcript per million mapped reads (FPKM). Genes were considered as DE when the absolute value of fold change was greater than 2 and *P* value was less than 0.05.

### Gene annotation analysis

Annotation analysis for dysregulated genes were conducted using KEGG (http://www.kegg.jp/), GO (http://www.geneontology.org/), Enzyme Commission (EC) (http://enzyme.expasy.org/), UniProt Knowledgebase (http://www.uniprot.org/help/uniprotkb/), Evolutionary Genealogy of Genes: Non-supervised Orthologous Groups (eggNOG) (http://eggnog.embl.de/version_3.0/) databases. Innate immune genes were annotated by InnateDB (https://www.innatedb.ca/) and Reactome Pathway database (http://www.reactome.org/).

### GO/KEGG enrichment analysis

GO and KEGG (www.kegg.jp/kegg/kegg1.html) enrichment analysis was performed using KOBAS database (http://kobas.cbi.pku.edu.cn/kobas3). In GO and KEGG enrichment analysis, p value was obtained by hypergeometric test and then corrected using the multiple hypothesis test of Benjamini and Hochberg (FDR correction method). *P* value after false discovery rate (FDR) correction < 0.05 represented that the terms were significantly enriched. KEGG enrichment analysis was performed as previously described^48–50^.

### Target prediction

Bioinformatics prediction analysis software miRanda was used to analyze the possible interactions between miRNAs and circRNAs or mRNAs.

### Venn analysis

Venn analysis was performed using the jvenn website (http://jvenn.toulouse.inra.fr/app/example.html)^51^.

### Interaction analysis

The interactions among proteins encoded by DE innate immune genes were analyzed through STRING: functional protein association networks (https://string-db.org/).

### Cell culture

DF-1 chicken embryo fibroblast cells (American Type Culture Collection, Manassas, VA, USA) were maintained in Dulbecco’s modified Eagle’s medium (DMEM, Thermo Scientific) containing 10% fetal bovine serum (Thermo Scientific) at 37 °C in a 5% CO_2_ atmosphere.

### Reagents and cell transfection

The INSIG1-specific small interference RNAs (si-INSIG1) and its negative control si-con were designed and synthesized from Shanghai GenePharma Co., Ltd. (Shanghai, China). The gga-miR-6631-5p mimic and NC mimic control, gga-miR-6631-5p inhibitor (in-miR-6631-5p) and its negative control in-NC were purchased from Thermo Scientific Co., Ltd. Cell transfection was performed using Lipofectamine 3000 Reagent (Thermo Scientific) following the instructions of manufacturer.

### RT‐qPCR assay

RNA was isolated and quantified as described above. cDNA first stands were synthesized from RNA template using the iScript cDNA Synthesis Kit (Bio-Rad, Hercules, CA, USA) and random primer (for mRNAs) or stem-loop gga-miR-6631-5p RT primer (5’-CTCAACTGGTGTCGTGGAGTCGGCAATTCAGTTGAGGCAGAACC-3’). Next, real-time quantitative PCR reaction was performed using ITaq Universal SYBR Green Super mix (Bio-Rad) and quantitative primers. Relative expression levels of gga-miR-6631-5p and mRNAs were calculated using the method of 2^−ΔΔCT^. GAPDH or 5S ribosomal RNA (5S rRNA) functioned as the endogenous inference to normalize the expression of mRNAs or gga-miR-6631-5p, respectively. Quantitative PCR primers were presented as follows: 5’-ACACTCCAGCTGGGGAAGAGAATGCTGTGG-3’ (forward) and 5’-TGGTGTCGTGGAGTCG-3’ (reverse) for gga-miR-6631-5p; 5’-CACCACAGCCACAGGATTAC-3’ (forward) and 5’-GAAGTCCCCAAAGTCACAGTC-3’ (reverse) for E2F transcription factor 1 (E2F1); 5’-CAGGAGGAGTGACCGTAGGA-3’ (forward) and 5’-CTTTCACACTGGCACCATGC-3’ (reverse) for insulin induced gene 1 (INSIG1); 5’-CACATCCTGGGAGCACTCTG-3’ (forward) and 5’-GTACTCTCCTCAGTGGGGGT-3’ (reverse) for HSPA (Hsp70) binding protein 1 (HSPBP1); 5’-GTGGAATTTGTGGCAGCACC-3’ (forward) and 5’-CTCCTTCTGCTCCCTCGTTG-3’ (reverse) for serpin family G member 1 (SERPING1); 5’-TCGATCTGTGCTTGGAGTGC-3’ (forward) and 5’-GTACCGCTTGGTGTCGAAGT-3’ (reverse) for proteasome 26S subunit, non-ATPase 11 (PSMD11); 5’-GGTAACGCCCGATCT-3’ (forward) and 5’-CGGTATTCCCAGGAGG-3’ (reverse) for 5S rRNA; 5’-GCGAGATGGTGAAAGTCGGA-3’ (forward) and 5’-TTCCCGTTCTCAGCCTTGAC-3’ (reverse) for GAPDH.

### Luciferase reporter assay

Partial fragment of INSIG1 3’UTR covering gga-miR-6631-5p binding sites was constructed into psiCHECK-2 vector by Hanbio Biotechnology Co., ltd. (Shanghai, China) and the recombinant plasmid was termed as INSIG1-wt reporter. INSIG1-mut reporter containing mutant gga-miR-6631-5p binding sites was also constructed by Hanbio Biotechnology Co., ltd. DF-1 cells were co-transfected with gga-miR-6631-5p mimic or NC mimic control and INSIG1-wt or INSIG1-mut reporter. At 48 h upon transfection, luciferase activities were measured through Dual-Luciferase Reporter Assay Kit (Promega, Madison, WI, USA).

### TCID_50_ (50% tissue culture infective dose) assay

DF-1 cells were transfected with corresponding miRNA mimic, miRNA inhibitor or siRNA, alone or in combination. At 24 h after transfection, DF-1 cells were infected with LaSota NDV at the multiplicity of infection (MOI) of 1. At 12, 24, 36, 48 h after infection, virus titers in cell supernatant were determined through TCID_50_ assay using Reed-Muench method.

### Statistics analysis

Statistics analysis was performed on GraphPad Prism 7 software (GraphPad Software, Inc., San Diego, CA, USA) with *P* < 0.05 as statistically significant. Difference between groups was compared using Student’s *T* test. Results were presented as mean ± standard deviation (SD).

### Ethical approval

All methods were carried out in accordance with relevant guidelines and regulations.

## Supplementary Information

Supplementary Information 1.

Supplementary Information 2.

Supplementary Information 3.

Supplementary Information 4.

Supplementary Information 5.

Supplementary Information 6.

Supplementary Information 7.

Supplementary Information 8.

Supplementary Information 9.

Supplementary Information 10.

Supplementary Information 11.

Supplementary Information 12.

Supplementary Information 13.

Supplementary Information 14.

Supplementary Information 15.

Supplementary Information 16.

Supplementary Information 17.

Supplementary Information 18.

Supplementary Information 19.

Supplementary Information 20.

Supplementary Information 21.

Supplementary Information 22.

Supplementary Information 23.

Supplementary Information 24.

Supplementary Information 25.

Supplementary Information 26.

## Data Availability

The data displayed in this manuscript is available from the corresponding author upon reasonable request.
